# The association between expectations of stigma and experiences of structural stigma in healthcare encounters among a cross-section of Canadians living with a mental illness or substance use disorder

**DOI:** 10.1371/journal.pmen.0000491

**Published:** 2026-03-03

**Authors:** Kai Yin Ernie Wong, Keith S. Dobson, Brooke Linden, Heather Stuart

**Affiliations:** 1 Department of Public Health Sciences, Queen’s University, Kingston, Ontario, Canada; 2 University of Calgary, Calgary, Alberta, Canada; PLOS: Public Library of Science, UNITED KINGDOM OF GREAT BRITAIN AND NORTHERN IRELAND

## Abstract

People living with mental illnesses or substance use disorders often anticipate being devalued or discriminated against by others (anticipatory stigma). These expectations can lead to heightened sensitivity to social rejection, hypervigilance, and overreactions to perceived threats. Healthcare settings are a common context for structural stigma, where negative organizational cultures and provider behaviours can compromise patient-provider interactions and quality of care, contributing to poorer health outcomes. Grounded in Modified labelling theory, this study examined whether higher expectations of stigma were associated with greater self-reported experiences of structural stigma during healthcare encounters. Data were drawn from a 2022 national sample of Canadians 18 years and older who reported a healthcare visit within the recall period (analytic n = 1509). Expectations of stigma and experiences of structural stigma in healthcare settings were measured using the Perceived Devaluation and Discrimination Scale and the Stigma Cultures in Healthcare Scale, respectively. Contrary to our hypotheses, experiences of structural stigma decreased as expectations of stigma increased. This association was only significant among males and in three out of the four models tested. Expectations of stigma related to a mental illness were associated with experiences of stigma during mental healthcare visits, and expectations of stigma related to a substance use disorder were associated with experiences of stigma during both mental and physical healthcare visits, controlling for the effects of age and estrangement from family and friends. These findings suggest that protective coping strategies, selective engagement with healthcare, and expectancy effects may shape the relationship between anticipated and experienced stigma, highlighting the complex interplay between individual psychological predispositions and structural healthcare environments.

## Introduction

People who experience mental illnesses or substance use disorders often expect to be stigmatized by others [1]. Modified labelling theory suggests that individuals internalize societal conceptions of mental illness early in life through the process of socialization [2]. These internalized beliefs develop into a lay theory about what it “means” to have a mental illness and shape individuals’ beliefs about how people with these labels are likely to be treated. In turn, these ideas often lead to “self-stigma,” or expectations of being rejected, devalued, or discriminated against if the individual themselves develops a mental illness. Originally conceptualized by Link [1] as “expectations of rejection,” this conceptual framework is consistent with the more modern concept of “anticipatory stigma,” which is prevalent in the extant literature (e.g., [3–5]).

Anticipatory stigma can influence both psychological wellbeing and healthcare-seeking behaviours [1]. High expectations of stigma may lead to rejection sensitivity, a psychological process whereby individuals learn to anticipate rejection due to previous experiences with overt or covert prejudice and discrimination [6–8]. Individuals with high rejection sensitivity are often hypervigilant for signs of rejection and overreactive to perceived threats [7]. In addition to lower self-esteem, individuals with higher expectations of devaluation or discrimination tend to demonstrate a greater avoidance of healthcare services [9–11]. This resistance to help-seeking has been linked in part to expectations of experiencing stigma during healthcare encounters. In fact, stigma from healthcare providers has been linked to numerous negative help seeking outcomes, including reluctance to seek care, early termination of treatment, poor therapeutic relationships, lower quality of care, and safety concerns [12].

Stigma experienced in healthcare settings often reflects broader processes of structural stigma. Stuart and Knaak define structural stigma in these settings as processes and interactions that are woven into the very fabric of healthcare organizations through structures such as policies, procedures, and practices expressed through client-provider interactions and quality of care [13]. Components of stigmatizing healthcare cultures described in the literature include negative attitudes and behaviours of healthcare providers towards those living with a mental illness, diagnostic overshadowing (the misattribution of physical health conditions to mental illnesses), paternalism that excludes clients from decision-making, and therapeutic pessimism (the belief that persons with mental illnesses have lower chances of recovery) [12,14,15]. A 2014 systematic review found that frequencies of discrimination reported by individuals living with a mental illness ranged from 16-44% in mental healthcare settings and 17–31% in physical healthcare settings [16]. More recently, Stuart and Knaak found that two thirds of a national sample of Canadians (n = 457) reported at least one stigmatizing experience in a healthcare setting, with 28% reporting six or more [13].

While previous literature has examined anticipatory stigma and structural stigma separately, few studies have investigated how these psychological predispositions interact with healthcare structures to influence patient experiences. The current study addresses this gap by exploring the relationship between expectations of stigma related to a mental illness or substance use disorder and self-reported experiences of structural stigma within a healthcare setting. By examining both individual-level expectations of stigma and structural experiences in healthcare, this study integrates both psychological and systemic perspectives on stigma, providing a more comprehensive understanding of its impact.

The goal of this study was to examine whether general expectations of stigma among individuals living with a mental illness or substance use disorder were associated with self-reported experiences of structural stigma during a recent healthcare experience. The objectives guiding this study were:

1) To explore if these associations differed based on whether the expectations of stigma were associated with a mental illness or a substance use disorder,
2) To explore whether these associations differed based on the purpose of the healthcare visit (e.g., for physical health problem vs. a mental health problem), and
3) To adjust for age, gender, and estrangement from family members and friends as potential confounders and/or effect modifiers.

We hypothesized that higher expectations of stigma, regardless of whether that stigma was related to a mental illness or a substance use disorder, would be associated with greater reported experiences of structural stigma in healthcare settings. Furthermore, we expected that those seeking care for a physical health problem would be less likely to report experiencing structural stigma compared to those seeking care for a mental health problem.

## Methods

### Ethics statement

This study was conducted in accordance with the Declaration of Helsinki and was approved by Queen’s University’s Health Sciences and Affiliated Teaching Hospitals Research Ethics Board (HSREB). Participant consent was not required due to the use of de-identified secondary data.

### Study design and sample

**Fig 1:**
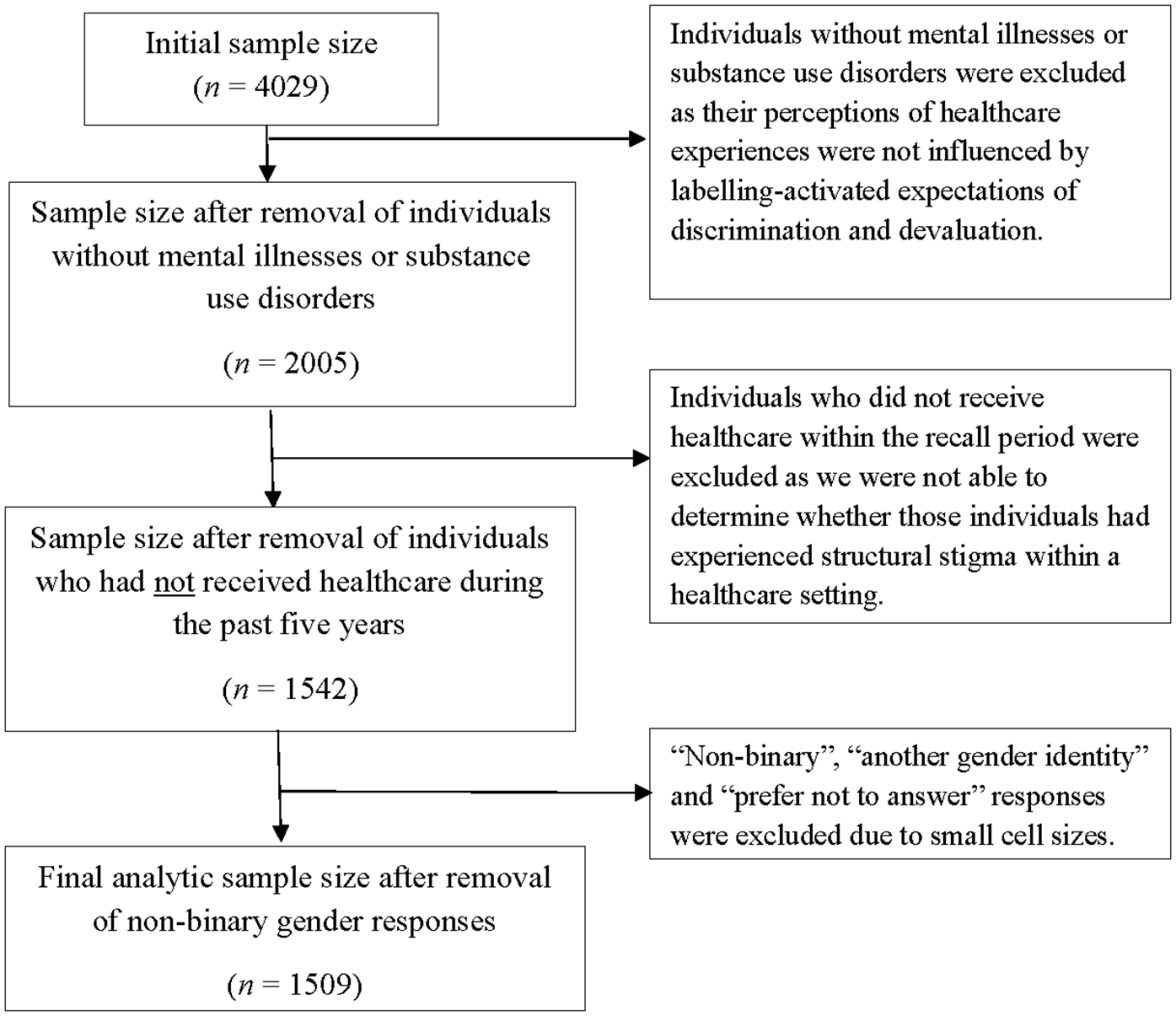
Flow chart showing the process of data abstraction.

This study involved a secondary analysis of cross-sectional data derived from a 2022 national stigma survey [17]. Data were collected from a large polling firm (Leger) and consisted of Canadians 18 years of age or older who reported having a healthcare experience within the last two years (n = 4029). The current study used a subset of these data (available in supplementary file S1 Data). De-identified data were first accessed for research purposes on May 16, 2022, following ethics clearance. Respondents were excluded if they did not report living with a mental or substance use disorder, had not received healthcare services within the recall period, or identified as having a non-binary gender identity (cell counts too small for analysis), resulting in a final analytic subsample of n = 1509 (see Fig 1).

### Sample size calculation

Because this study involved a secondary analysis of an existing national survey dataset, no *a priori* sample size calculation was conducted. Power was assessed post hoc by estimating the minimum detectable effect size given the range of analytic sample sizes across statistical models. Assuming a two-sided α of 0.05, 80–90% power, equal proportions of exposed and unexposed participants, and plausible estimates of outcome prevalence based on prior Canadian population data, minimum detectable odds ratios for the full analytic subsample (n = 1509) ranged from approximately 1.29 to 1.34. Corresponding minimum detectable risk ratios ranged from approximately 1.07 to 1.09. Overall, these estimates suggest the study was sufficiently powered to detect moderate associations between stigma expectations and healthcare experience outcomes.

### Measures

***Expectations of stigma*** *(exposure)* were measured using the 12-item Perceived Devaluation and Discrimination Scale (PDD) [1]. This scale measures the extent to which a respondent thinks that “most people” would devalue or discriminate against someone with a mental or substance use disorder. Reponses are provided on a 4-point agreement scale ranging from ‘strongly agree’ to ‘strongly disagree.’ Aggregate composite scores range from 12 to 48, where higher scores indicated greater expectations of stigma. The PDD is the most used scale to evaluate expectations of stigma, demonstrating strong evidence of validity and internal consistency (reliability)[1]. The tool has been translated and validated into multiple languages, including German, Swedish, and Chinese, demonstrating strong reliability and validity across contexts [18].

We examined expectations of stigma separately for mental illness and substance use disorder. The PDDS was posed to respondents twice: first, in the context of a substance use disorder (e.g., “most people would think less of a person who has been treated for a **substance use disorder**”), and second, in the context of a mental illness (e.g., “most people would think less of a person who has been treated for a **mental illness**”). This resulted in two exposure variables for our analyses: 1) expectations of mental illness-related stigma and 2) expectations of substance use-related stigma. The coefficient alphas for the composite PDDS scores were α = 0.93 for substance use-related stigma (M = 35.4, SD = 7.0) and α = 0.92 for mental illness-related stigma (M = 34.0, SD = 7.8), indicating high internal reliability.

***Structural stigma*** *(outcome)* was measured using seven items from the Stigma Cultures in Healthcare Scale (SCHS) [13]. This scale measures the extent to which healthcare cultures are experienced as stigmatizing by service users on a 4-point agreement scale ranging from ‘strongly agree’ to ‘strongly disagree.’ The original, 23-item scale has shown strong evidence of both validity and reliability. We used a subset of seven items from the scale for the current study, selecting those that captured whether respondents felt that: they were taken seriously, their needs were met, they were paid attention to, they had a choice, they felt supported, instructions were clear, and their concerns were met [13]. Aggregated, composite scores for this subscale ranged from 7 to 28, where higher scores indicated more structural stigma experiences in a healthcare setting.

Thinking about their most recent healthcare experience within the past two years, respondents were asked to respond to this set of questions twice: first, for a physical health problem, and second, for a mental health problem. As with our exposure, this resulted in two outcome variables for our analyses: 1) structural stigma experienced during a healthcare visit for a physical health problem, and 2) structural stigma experienced during a healthcare visit for a mental health problem. These outcome variables were analyzed separately. The coefficient alphas for the composite subscale scores in the current sample were α = 0.90 (M = 13.8, SD = 4.9) for a healthcare visit for a physical health problem, and α = 0.92 (M = 13.9, SD = 5.2) for a mental health problem, indicating high internal reliability.

**Estrangement from friends/family** *(covariate)* was measured using two individual survey items that asked respondents to indicate their level of agreement with the statement, “I experienced rejection or estrangement from my family/friends.” The item was rated on a 4-point scale ranging from ‘strongly agree’ to ‘strongly disagree’. These were operationalized as separate, binary variables (e.g., estrangement from friends, and estrangement from family) where 1 = participants endorsing *any* rejection/estrangement items (agreement) and 0 = *no* endorsement of any rejection/estrangement (disagreement).

**Age** *(covariate)* was included in the multivariate models as a continuous variable, measured in years.

**Gender** *(effect modifier)* was operationalized as a binary variable (male/female). Though additional gender identity categories were included on the original survey (e.g., non-binary, other), low cell counts in our analytic subsample did not allow us to analyze this group of individuals. Given that gender is routinely identified in the literature as an effect modifier of relationships between various exposures and mental health outcomes, we explored this variable as a potential effect modifier of the relationship of interest.

### Statistical analysis

Analyses were conducted to address each study objective and hypothesis. Descriptive statistics were first calculated to determine the nature of the sample and the distribution of the variables for analysis. Generalized linear regression models were then specified to estimate the associations between expectations of stigma and experiences of structural stigma during a recent healthcare visit.

**Fig 2:**
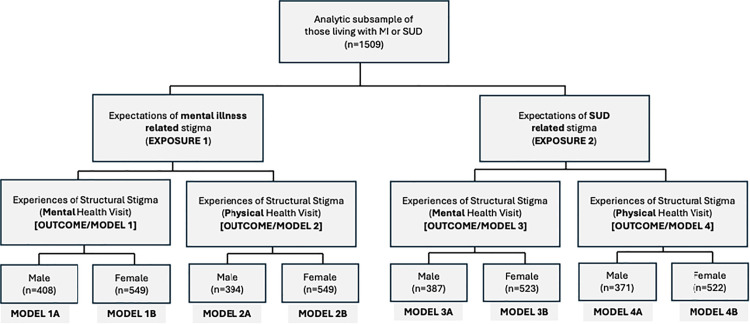
Stratification of generalized linear models.

**Fig 3:**
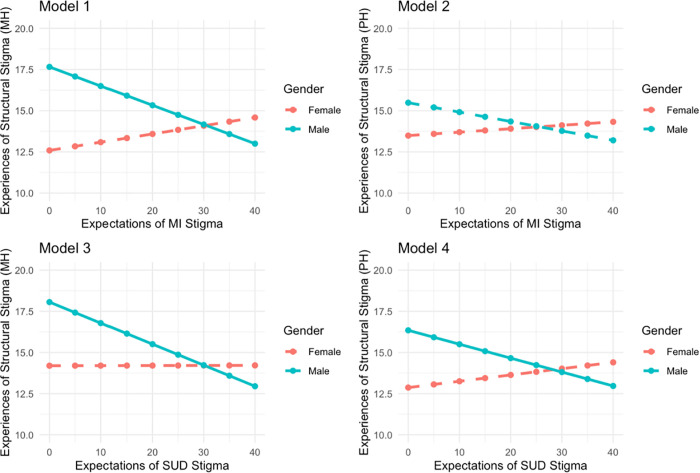
Predicted Values Plots for all Models, Stratified by Gender.

Because our analysis included two exposure variables and two outcome variables of interest, a total of four models were specified. After confirming the presence of effect modification, all models were further stratified by gender with male models presented as “A” and female models presented as “B.” The diagram in Fig 2 shows the stratification process. Examination of tolerance and Variance Inflation Factor (VIF) values revealed no multicollinearity issues in any of the models. Associations were then visually depicted using predicted value plots stratified by gender (Fig 3). Models were specified using a complete case analysis approach to missing data, meaning any respondent with complete information for the variables relevant to an individual model were included in the analysis. Analytic sample sizes varied across models due to outcome-specific missingness (see S1 Text for missing data). All statistical analyses were completed in R Version 4.4.3.

## Results

### Sample

Table 1 summarizes sample characteristics stratified by gender, reflecting planned effect modification analyses. The overall analytic sample consisted of n = 593 male and n = 916 female respondents. The average age of participants was 44.9 years (*SD* = 14.9) for males, and 42.2 years (*SD* = 14.8) for females (p < 0.001), with an overall range of 20–87 years. Approximately 45% of respondents reported estrangement from friends due to their mental illness or substance use disorder, and approximately 40% reported estrangement from family, with no significant differences observed by gender.

**Table 1:** *S*ample Characteristics (n = 1,509).

Categorical Variables	Male (n = 593)	Female (n = 916)	
	n (%)	n (%)	p
Experienced Estrangement from Friends			
Yes	248 (44.2)	404 (46.6)	0.406
No	313 (55.8)	463 (53.4)	
Experienced Estrangement from Family			
Yes	228 (39.7)	328 (37.7)	0.484
No	346 (60.3)	541 (62.3)	
**Continuous Variables**	**Mean (SD)**	**Mean (SD)**	**p**
Age	44.90 (14.91)	42.22 (14.82)	**<0.001**
Expectations of mental illness-related stigma	34.56 (7.81)	33.59 (7.77)	**0.046**
Expectations of substance use-related stigma	35.13 (7.15)	35.61 (6.89)	0.283
Experiences of Structural Stigma (Mental HC)	13.60 (4.79)	14.09 (5.51)	0.087
Experiences of Structural Stigma (Physical HC)	13.51 (4.43)	13.97 (5.11)	0.087

*Note.* HC = healthcare; chi-square (X^2^, categorical) and t-tests (t) run to assess sample differences; significant findings are bolded.

Average scores for expectations of mental illness-related stigma were significantly lower among females, though the difference was small and of borderline statistical significance (p = 0.046). There was no significant difference in average scores for expectations of substance use-related stigma by gender. Similarly, experiences of structural stigma for healthcare visits related to mental or physical health problems were not statistically different by gender.

### Expectations of mental illness-related stigma and experiences of structural stigma

Tables 2 and 3 summarize the results of generalized linear regression models examining the associations between expectations of mental illness-related stigma and experiences of structural stigma during a healthcare visit for a mental health problem (Model 1, Table 2) or physical health problem (Model 2, Table 3). Models were further stratified by gender (e.g., results for males displayed in Model 1A, results for females displayed in Model 1B).

**Table 2:** Model 1 - Gender Stratified Generalized Linear Regression Analysis of the Associations between Expectations of Mental Illness-related Stigma and Experiences of Structural Stigma During a Healthcare Visit for a Mental Health Problem.

Healthcare Visit for a Mental Health Problem	*Model 1 A* *Males (n = 408)*			*Model 1B* *Females (n = 549)*		
	*B*	SE	*p*	*B*	SE	*p*
(Constant)	**16.730**	**1.336**	**<.0001**	**13.201**	**1.304**	**<.0001**
Expectations of mental illness-related stigma	**-0.116**	**0.035**	**0.001**	0.050	0.033	0.13
Estrangement from Friends	**1.240**	**0.583**	**0.03**	**1.183**	**0.525**	**0.02**
Estrangement from Family	0.378	0.576	0.51	**1.151**	**0.530**	**0.03**
Age	0.005	0.016	0.75	**-0.038**	**0.017**	**0.02**

*Note.* B = unstandardized regression coefficient, SE = standard error. Significant findings are bolded.

**Table 3:** Model 2 - Gender Stratified Generalized Linear Regression Analysis of the Associations between Expectations of Mental Illness-related Stigma and Experiences of Structural Stigma During a Healthcare Visit for a Physical Health Problem.

Healthcare Visit for a Physical Health Problem	Model 2A Males (n = 394)			Model 2B Females (n = 549)		
	*B*	SE	*p*	*B*	SE	*p*
(Constant)	**14.694**	**1.240**	**<.0001**	**14.307**	**1.204**	**<.0001**
Expectations of mental illness-related stigma	-0.057	0.033	0.08	0.021	0.030	0.49
Estrangement from Friends	**1.422**	**0.544**	**0.01**	**1.062**	**0.493**	**0.03**
Estrangement from Family	-0.722	0.534	0.18	**1.144**	**0.494**	**0.02**
Age	0.010	0.015	0.50	**-0.041**	**0.015**	**0.01**

*Note.* B = unstandardized regression coefficient, SE = standard error. Significant findings are bolded.

Model 1 revealed a statistically significant association between expectations of mental illness-related stigma and experiences of structural stigma during a healthcare visit for a mental health problem. However, this relationship was only significant among men, and in a direction contrary to our hypothesis. For every one-unit increase in expectations of mental illness-related stigma, the model showed a significant decrease in reported experiences of structural stigma during a healthcare visit for a mental health problem (B -0.116, p = 0.001), controlling for the effects of age and estrangement from family and friends.

In Model 2, the association between expectations of mental illness-related stigma and experiences of structural stigma during a healthcare visit for a **physical** health problem was not statistically significant for either gender.

### Expectations of substance use-related stigma and experiences of structural stigma

Tables 4 and 5 summarize the results of the next two generalized linear regression analyses, with models this time exploring the associations between expectations of substance use-related stigma and experiences of structural stigma during a healthcare visit for a mental health problem (Model 3, Table 4), or a physical health problem (Model 4, Table 5). Models were stratified by gender.

**Table 4:** Model 3 - Gender Stratified Generalized Linear Regression Analysis of the Associations between Expectations of Substance Use-related Stigma and Experiences of Structural Stigma During a Healthcare Visit for a Mental Health Problem.

Healthcare Visit for a Mental Health Problem	Model 3A Males (n = 387)			Model 3B Females (n = 523)		
	*B*	SE	*p*	*B*	SE	*p*
(Constant)	**18.321**	**1.419**	**<.0001**	**14.573**	**1.549**	**<.0001**
Expectations of substance use-related stigma	**-0.128**	**0.038**	**0.001**	0.0005	0.038	0.99
Estrangement from Friends	1.072	0.578	0.06	0.905	0.542	0.09
Estrangement from Family	-0.386	0.560	0.49	**1.700**	**0.534**	**0.001**
Age	-0.013	0.016	0.42	**-0.034**	**0.017**	**0.05**

*Note. B* = unstandardized regression coefficient, SE = standard error. Significant findings are bolded.

**Table 5:** Model 4 - Gender-Stratified Generalized Linear Regression Analyses of the Associations between Expectations of Substance Use-related Stigma and Experiences of Structural Stigma During a Healthcare Visit for a Physical Health Problem.

Healthcare Visit for a Physical Health Problem	Model 4A Males (n = 371)			Model 4B Females (n = 522)		
	*B*	SE	*p*	*B*	SE	*p*
(Constant)	**16.249**	**1.314**	**<.0001**	**13.866**	**1.446**	**<.0001**
Expectations of substance use-related stigma	**-0.084**	**0.035**	**0.01**	0.038	0.035	0.27
Estrangement from Friends	**1.513**	**0.534**	**0.005**	0.895	0.500	0.07
Estrangement from Family	**-1.180**	**0.513**	**0.02**	**1.041**	**0.493**	**0.03**
Age	-0.002	0.015	0.88	**-0.043**	**0.016**	**0.01**

*Note. B* = unstandardized regression coefficient, SE = standard error. Significant findings are bolded.

Model 3 revealed a statistically significant association between expectations of substance use-related stigma and experiences of structural stigma during a healthcare visit for a mental health problem. As in Model 1, this relationship was again only significant among men, and negative in direction. For every one-unit increase in expectations of substance use-related stigma, the model showed a significant decrease in reported experiences of structural stigma during a healthcare visit for a mental health problem (B -0.128, p = 0.001), controlling for the effects of age and estrangement from family and friends. No significant association was observed among females.

Model 4 revealed findings similar to that of Model 3. There was a statistically significant, negative association between expectations of substance use-related stigma and experiences of structural stigma during a healthcare visit for a physical health problem, but only among males. Again, no significant association was observed among females.

Fig 3 shows the predicted values plots for all models, illustrating the inverse associations between expectations of stigma and reported experiences of structural stigma in healthcare settings among males across models. Statistically significant decreases were noted in three models (depicted by the solid lines in Models 1, 3, and 4) with the largest decreases occurring in experiences of structural stigma during healthcare visits for a mental health problem.

## Discussion

This study examined the association between general expectations of stigma among individuals living with a mental illness or substance use disorder and self-reported experiences of structural stigma during a recent healthcare experience for either a mental or physical health problem. The sample consisted of a large group of Canadians with a self-reported mental illness or substance use disorder. Contrary to our hypothesis, the frequency of structural stigma experiences in healthcare settings did not increase as expectations of stigma increased. Rather, experiences of structural stigma significantly decreased as stigma expectations increased. However, this association was statistically significant only for males and in three of the four models tested. No significant associations were observed among female respondents. For males, expectations of stigma related to a mental illness were significantly associated with experiences of structural stigma during a mental healthcare visit. Additionally, expectations of stigma related to a substance use disorder were significantly associated with experiences of structural stigma during healthcare visits for both mental and physical health problems. All models controlled for the effects of age and estrangement from family and friends.

Although we hypothesized that greater expectations of stigma would predict higher perceived structural stigma, the opposite was observed. This counterintuitive finding may reflect protective coping strategies, selective engagement with healthcare, or differentiation between anticipatory beliefs and actual experiences. One possible explanation lies in *Expectancy Violation Theory* [19], which suggests that when individuals anticipate negative treatment but instead encounter neutral or positive interactions, this discrepancy can enhance their perception of the experience. For example, research suggests that males often hold more pessimistic expectations about mental healthcare and perceive higher levels of stigma due to social norms or past experiences [20]. The absence of expected stigma may then register as a positive violation, reducing their perceived experiences of structural stigma. The contrast effect proposed by expectancy violation may further amplify this dynamic, such that even a moderately respectful or neutral interaction is experienced as favorable when initial expectations are low.

Another possibility for the current results is that people may simply anticipate stigma more often than they experience it. For example, Angermeyer and colleagues [3] found that psychiatric inpatients (n = 209) anticipated stigma more frequently than they experienced it, and correlations between anticipated and experienced stigma were small. Though primarily focusing on interpersonal stigma among 732 people treated for schizophrenia in 17 countries, Thornicroft et al. [21] also noted a discrepancy between the proportion of the sample that anticipated, rather than experienced stigma. For example, 69% of the sample anticipated discrimination with respect to intimate relationships, but over half had not experienced it. They also noted that experienced discrimination scores varied among countries, but anticipated discrimination scores did not.

Modified labeling theory suggests that individuals who internalize societal stigma about mental illness develop expectations of rejection that shape their behaviour and social interactions [22]. These expectations may lead individuals to adopt protective strategies intended to mitigate anticipated discrimination and rejection, such as emotional disengagement, concealment of stigmatized attributes, or withholding of information from healthcare providers. These strategies may in turn alter both the clinical encounters and the patient’s evaluation of those encounters. Modified labeling theory further suggests that these coping mechanisms may unintentionally reinforce social isolation and exacerbate the negative consequences of stigma by limiting opportunities for positive interpersonal feedback and support. Moreover, individuals who expect stigma may selectively seek care only in “safe” or affirming environments or delay healthcare altogether until a trusted provider is available to reduce their exposure to structural stigma. This selective engagement may contribute to the counterintuitive finding that higher expectations of stigma are associated with lower reported experiences of structural stigma, as these individuals effectively avoid settings where stigma is more likely to be encountered. Modified labelling theory also acknowledges that stigma is a dynamic process shaped by social context, suggesting that the relationship between expectations and experiences of stigma may shift over time and differ by social identity factors such as gender. Future research should explore how these protective and avoidance behaviours evolve and interact with healthcare system factors to improve our understanding of how expectations of stigma translate into healthcare experiences and outcomes for diverse populations.

## Study limitations and future directions

Several limitations should be considered when interpreting the findings of this study. First, the data were derived from a self-report questionnaire, which may be subject to the effects of both recall bias and social desirability bias. Given the sensitive nature of both mental illnesses and substance use disorders, participants may have underreported their experiences of mental ill health, as well as discrimination or structural barriers they may have experienced. This issue is particularly relevant in healthcare contexts, where individuals may feel pressured to appear compliant or avoid attributing negative experiences to systemic factors. Replication of the study using alternative forms of data collection, such as interviews, is recommended.

Second, the study may have been affected by measurement bias due to potential misalignment between our exposure and outcome measures. Expectations of stigma captures generalized cumulative beliefs about how individuals with mental illnesses or substance use disorders expect to be treated. In contrast, our measure of structural stigma reflected self-reported experiences during a single recent healthcare visit. This difference between generalized beliefs and event-specific experiences may have resulted in some misclassification error, particularly if participants with high expectations of stigma encountered a neutral or positive interaction that did not reflect their broader views. Additionally, structural stigma is often systemic and institutionalized and may not be easily perceived or recognized within the context of a single encounter, further complicating measurement accuracy.

Third, the sample may be subject to selection bias. Survey-based research related to mental health tends to disproportionately attract individuals who are more likely to engage with research opportunities and may have greater access to healthcare. As a result, individuals experiencing the most severe forms of mental illness, the most acute forms of stigma, or the highest degrees of structural marginalization are likely to be underrepresented in this sample. If valid, this pattern may lead to an underestimation of both expectations of stigma and structural stigma experiences within healthcare settings among the broader population. Repetition of the current study methods with more representative samples may be able to mitigate this issue.

Fourth, due to small cell counts, our analysis was restricted to individuals who identified only as either male or female. This is a significant limitation as research consistently shows that non-binary and other gender-diverse individuals often report worse mental health outcomes and more negative healthcare experiences compared to cisgender peers [23–25]. Future research should prioritize the analysis of diverse gender identities to better understand the intersection between gender identity, anticipatory stigma, and structural stigma during healthcare encounters within these populations.

Fifth, it is not clear whether expectations of stigma at the population level (e.g., generalized social beliefs) correspond to individual expectations that one will be personally stigmatized within a healthcare setting. Without an available measure of individual treatment expectations, defined as whether someone would anticipate that they would be treated negatively or receive poorer quality of care because of their mental illness, we were unable to assess this construct. The role of individual-level treatment expectations in shaping healthcare experiences is only beginning to be recognized. According to Levenig et al. [26], for example, treatment expectations are shaped by information obtained through various sources, such as interactions with healthcare providers and the media, and are powerful enough to modulate pain and recovery experiences. It is possible that treatment expectations may also shape experiences of structural stigma in healthcare encounters. Patient expectations about processes of care may create a self-fulfilling prophecy. Future research examining factors associated with experiences of structural stigma would benefit from the use of a direct measure of treatment expectations.

Finally, due to the cross-sectional nature of the data, we are unable to determine causality or temporal relationships. Future research would benefit from the use of longitudinal designs that might capture more dynamic relationships between expectations of stigma and experiences of structural stigma in healthcare settings over time.

## Supporting information

S1 Text Sample missingness by gender. (DOC)

S1 Data Raw data. (CSV)
