# Supplementary material for: The association between expectations of stigma and experiences of structural stigma in healthcare encounters among a cross-section of Canadians living with a mental illness or substance use disorder
Source: PLOS Ment Health. 2026 Mar 3;3(3):e0000491. doi: 10.1371/journal.pmen.0000491 (PMC12956111; doi:10.1371/journal.pmen.0000491)
Supplement: S1 Text — (DOC) [file pmen.0000491.s001.doc]

**Supplementary File**

**Sample Missingness by Gender**

| **Variable** | **Male (%)**  **(n=593)** | **Female (%)**  **(n=916)** | **Total Missing (%)**  **(n=1509)** |
| --- | --- | --- | --- |
| Mental HC | 32 (5.4) | 85 (9.3) | 117 (7.7) |
| Physical HC | 53 (8.9) | 99 (10.8) | 152 (10.1) |
| Expectations of stigma (SUD) | 185 (31.2) | 343 (37.4) | 528 (35.0) |
| Expectations of stigma (MI) | 156 (26.3) | 294 (32.1) | 450 (29.8) |
| Estrangement from friends | 32 (5.4) | 49 (5.3) | 81 (5.4) |
| Estrangement from family | 19 (3.2) | 47 (5.1) | 66 (4.4) |
| Age | 0 | 0 | 0 |

*Note.* MI = mental illness; SUD = substance use disorder; HC = healthcare
